# Brazilin from *Caesalpinia sappan* inhibits viral infection against PRRSV via CD163^ΔSRCR5^ MARC-145 cells: an in silico and in vitro studies

**DOI:** 10.1038/s41598-022-26206-x

**Published:** 2022-12-14

**Authors:** Chaiwat Arjin, Suriya Tateing, Nuttha Potapohn, Jirapat Arunorat, Kidsadagon Pringproa, Chompunut Lumsangkul, Mintra Seel-audom, Warintorn Ruksiriwanich, Korawan Sringarm

**Affiliations:** 1 Department of Animal and Aquatic Sciences, Faculty of Agriculture, Chiang Mai University, Chiang Mai, 50200 Thailand; 2 Department of Plant and Soil Sciences, Faculty of Agriculture, Chiang Mai University, Chiang Mai, 50200 Thailand; 3 Department of Veterinary Bioscience and Veterinary Public Health, Faculty of Veterinary Medicine, Chiang Mai University, Chiang Mai, 50100 Thailand; 4 Department of Pharmaceutical Sciences, Faculty of Pharmacy, Chiang Mai University, Chiang Mai, 50200 Thailand; 5 Cluster of Research and Development of Pharmaceutical and Natural Products Innovation for Human or Animal, Chiang Mai University, Chiang Mai, 50200 Thailand

**Keywords:** Virology, Virtual drug screening

## Abstract

This research aimed to identify bioactive compounds from *Caesalpinia sappan* extract that function as novel porcine reproductive and respiratory syndrome virus (PRRSV) infection inhibitors by computational molecular screening. We obtained a set of small-molecule compounds predicted to target the scavenger receptor cysteine-rich domain 5 (SRCR5) of CD163. In addition, the functions of positive hits were assessed and verified utilizing an in vitro antiviral activity assay with PRRSV-infected MARC-145 cells. Combining molecular docking with the results of binding affinity and ligand conformation, it was found that brazilin had the highest binding energy with the SRCR5 receptor compared to catechin and epicatechin (− 5.8, − 5.5, and − 5.1 kcal/mol, respectively). In terms of molecular mechanics, the binding free energy between the SRCR5 receptor was − 15.71 kcal/mol based on the Poisson-Boltzmann surface area of brazilin. In addition, PRRSV infection in MARC-145 cells was significantly inhibited by brazilin compared to the control (virus titer, 4.10 vs. 9.25 TCID_50_/mL, respectively). Moreover, brazilin successfully limited the number of PRRSV RNA copies in MARC-145 cells as determined by RT-qPCR. By inhibiting the PRRSV-CD163 interaction with brazilin from *Caesalpinia sappan*, it may be possible to prevent PRRSV infection in pigs, as suggested by this research.

## Introduction

Porcine reproductive and respiratory syndrome virus (PRRSV) is widespread in the majority of pig-producing nations and causes substantial economic losses for the worldwide swine industry^1^. PRRSV infection typically causes a condition manifested by reproductive failure in sows and respiratory infections in growing pigs^2^. PRRSV is an enveloped, single-stranded, positive-sense RNA virus that is a member of the Arteriviridae family (order Nidovirales), which also includes the horse arteritis virus, mouse lactate dehydrogenase-elevating virus, and simian hemorrhagic fever virus^3^. PRRSV shows a very narrow host cell tropism, infecting only specific porcine macrophage subsets^4^. Based on productive PRRSV infection, pulmonary alveolar macrophages (PAMs) are the primary target cells. PRRSV entrance mediators include two macrophage-specific molecules: siglec sialoadhesin and scavenger receptor CD163^5^. CD163 has been determined to be the major receptor mediating viral internalization and disassembly^3^. However, the green monkey kidney cell lines (MA-104 and MARC-145) also express CD163 and are suitable for studying PRRSV infection in vitro. CD163, a macrophage-specific protein in the scavenger receptor cysteine-rich (SRCR) superfamily, functions as a cellular receptor for porcine reproductive and respiratory syndrome virus (PRRSV)^6^. The extracellular portion of CD163 forms a pearl-on-a-string structure of nine scavenger receptor cysteine-rich (SRCR) domains and is anchored by a single transmembrane segment and a short cytoplasmic domain^4,7^. Further research revealed that the SRCR5 domain of CD163's extracellular region plays a crucial role in PRRSV infection^8^. The removal of SRCR5 from CD163 confers complete resistance to PRRSV in pigs^4,9^. Consequently, SRCR5 in porcine CD163 is one of the most promising molecular targets for preventing PRRSV infection^10^.

Although vaccinations have been used to prevent this disease, several vaccine types, including modified live-virus vaccines, have failed to eradicate the virus and do not confer complete immunity against heterologous infections^11^. Currently, there are no effective commercial drugs available to control PRRSV infection^12^. Researchers are eager to discover a method for controlling PRRSV. Several compounds have been utilized as anti-PRRSV agents, such as dipotassium glycyrrhetate^13^, N-acetylpenicillamine^14^, and tetrahydroaltersolanol C^15^. However, the anti-PRRSV activity of these agents appears to be nonspecific, as some of them limit the reproduction of multiple different viruses. Consequently, these medicines are not yet suitable for use in anti-PRRSV therapy^16^. An alternative approach is the investigation of phytochemical therapeutics. Based on prior research, the crude extract of *Caesalpinia sappan* (CS) has the potential to inhibit viral infection and replication in vitro against PRRSV in the MARC-145 cell line^17^. CS is a member of the Leguminosae family, and it is cultivated in tropical Asian locations such as southern China, India, Myanmar, Sri Lanka, and Thailand. The dried heartwood of CS has been utilized for centuries in traditional oriental remedies, including Ayurveda and Traditional Chinese Medicine^18^. A CS heartwood decoction is utilized as an anti-inflammatory drug for the treatment of traumatic disease and arthritis in northern Thailand, particularly in the Chiang Mai, Nan, and Lampang provinces^19^. As a result of our earlier research, we discovered bioactive groups in CS crude extract that have significant antiviral activity against PRRSV, such as brazilin, naringenin, catechin and epicatechin^11^. However, we were only able to isolate three compounds in our investigation (brazilin, catechin, and epicatechin). Brazilin is the main homoisoflavonoid constituent found in CS heartwood^20^ and possesses various bioactive properties, such as antioxidant^19,21^, antibacterial^22,23^, and antiviral^24^ activities. No evidence of antiviral activity against PRRSV is currently available in the Brazilian literature; therefore further investigation is warranted. However, the field of compound discovery has been widened by recent advances in computational biology approaches. As a result of advancements in structural biology, computer science, and bioinformatics, it is now simple to identify putative molecules for experimental evaluation by screening a large database^10^. It is now well accepted that molecular docking is an important tool for the development and screening of novel antiviral compounds, especially those caused by viruses^25^. This approach facilitates the selection of the most effective antiviral compound. Thus, the goal of this investigation was to identify bioactive compounds from CS extract as novel inhibitors of PRRSV infection by targeting the SRCR5 receptor. To find bioactive chemicals in CS extracts, researchers employed structure-based virtual screening, molecular dynamics (MD), and molecular mechanics (MM) simulations. Thus, in vitro procedures can be used to verify the computational conclusions.

## Results

### Molecular docking studies

Three natural compounds (brazilin, catechin and epicatechin), as major compounds of *Caesalpinia sappan* L., were docked into the binding site of the SRCR5 protein receptor with binding energy values of − 5.8, − 5.5, and − 5.1 kcal/mol, respectively. Based on the binding affinity and ligand conformation results, brazilin showed the best binding energy, followed by catechin and epicatechin (Fig. S1). Brazilin interacted with Asp505, Phe506, and Ala510 with hydrophobic interactions as well as Asp505, Ser507, Glu543, Phe544, and Gln545 with hydrogen bonding. Catechin interacted with Ser504, Ser507 and Gln545 as hydrophobic interactions as well as Asp505, Phe506, Glu543 and Cys546 with hydrogen bonding. Epicatechin interacted with Gln545 via hydrophobic interactions as well as Ser504, Ser507, Glu543, and Cys546 via hydrogen bonding. The best poses of each ligand were used as the initial structure for further studying the molecular dynamics simulations of SRCR5-inhibitor complexes.

### Molecular dynamics simulation studies

#### Molecular dynamics analysis

**Figure 1:**
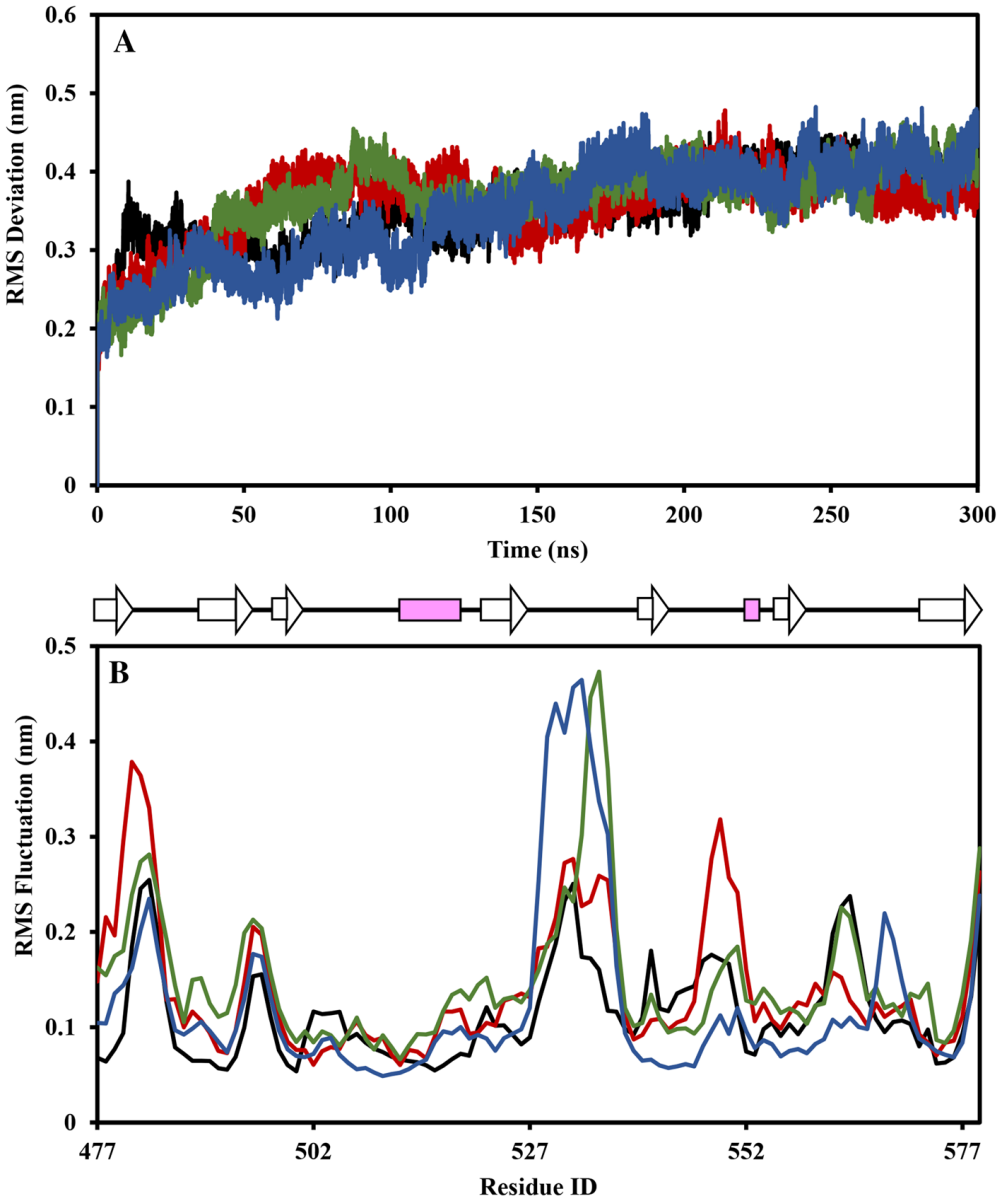
RMSD (**A**) and RMSF (**B**) of unbound and bound SRCR5 forms with three inhibitors: (black represents unbound SRCR5, red represents SRCR5-brazilin, green represents SRCR5-catechin, blue represents SRCR5-epicatechin).

MD simulations of all complexes were performed for 300 ns to determine the dynamics of the SRCR5 receptor when bound with different natural compounds and compared with those of the unbound SRCR5 receptor. Overall structural dynamics of the protein receptor were observed. The RMSD and RMSF values obtained from the MD trajectories of unbound and bound forms in SRCR5-inhibitor complexes are presented (Fig. 1). It was observed that the RMSD of the bound forms was initially higher than that of the unbound form of the SRCR5 receptor. However, after 100 ns, the RMSDs in all complexes showed nearly similar trends (Fig. 1A). The RMSF profiles were determined for the position of fluctuation, and it was observed that in each case, the highest fluctuation occurred at the point of bound forms (Fig. 1B). It was also observed that catechin and epicatechin compounds bound to the SRCR5 receptor showed higher RMSF to residues 530–540. Brazilin bound to the SRCR5 receptor affected neighboring residues, resulting in a higher RMSF up to residue 552. Furthermore, the RMSF values for the bound forms were lower at residue 560, indicating a loss of some flexibility.

#### Molecular overlay of SRCR5 with the three inhibitors

**Figure 2:**
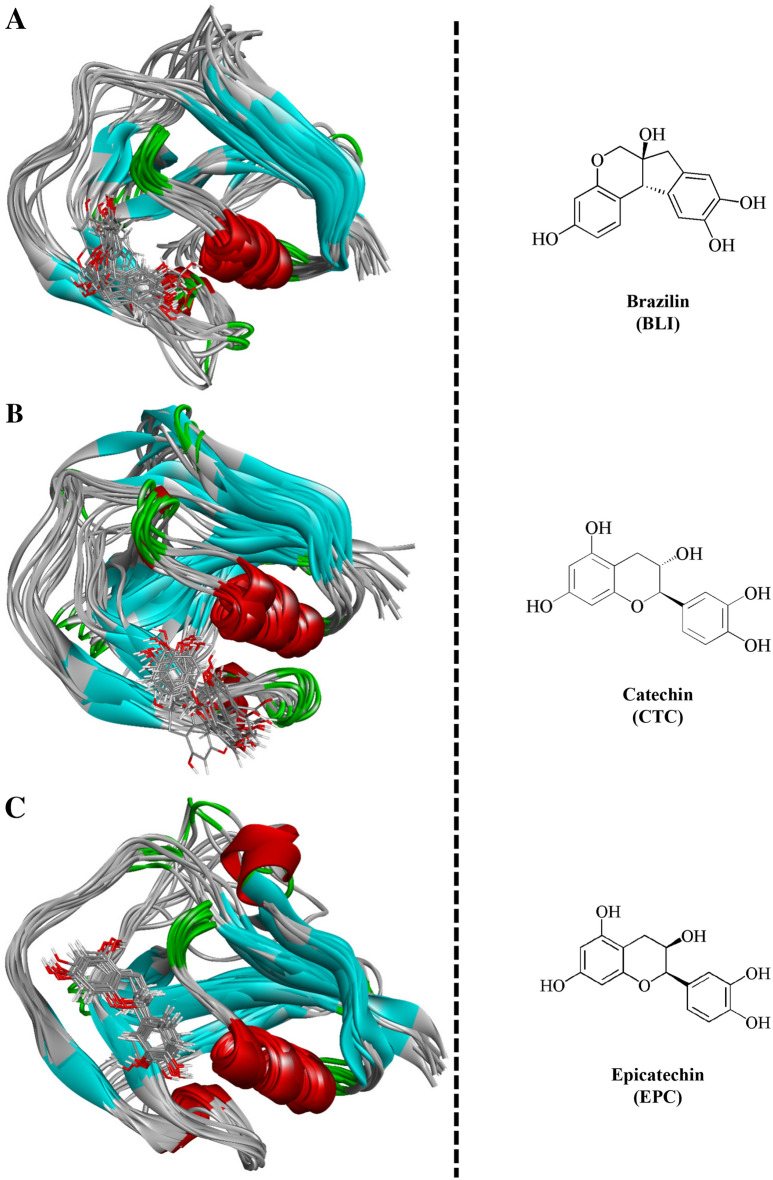
Molecular overlay of SRCR5-inhibitor complexes: (**A**) brazilin (**B**) catechin and (**C**) epicatechin.

To study the dynamics of SRCR5 receptor binding with the different inhibitors, each of the last snapshots, 100–300 ns, of the MD trajectories were extracted and separated into protein‒ligand conformations with an interval time of 20 ns (Fig. 2). Molecular overlay, a module of Discovery Studio Visualizer, was used to allow the molecules to be superimposed based on the similar functions of the protein structures. The molecular overlay results suggested that the structures of brazilin and epicatechin remained stable on the SRCR5 receptor, while catechin showed significant deviation from brazilin and epicatechin.

**Figure 3:**
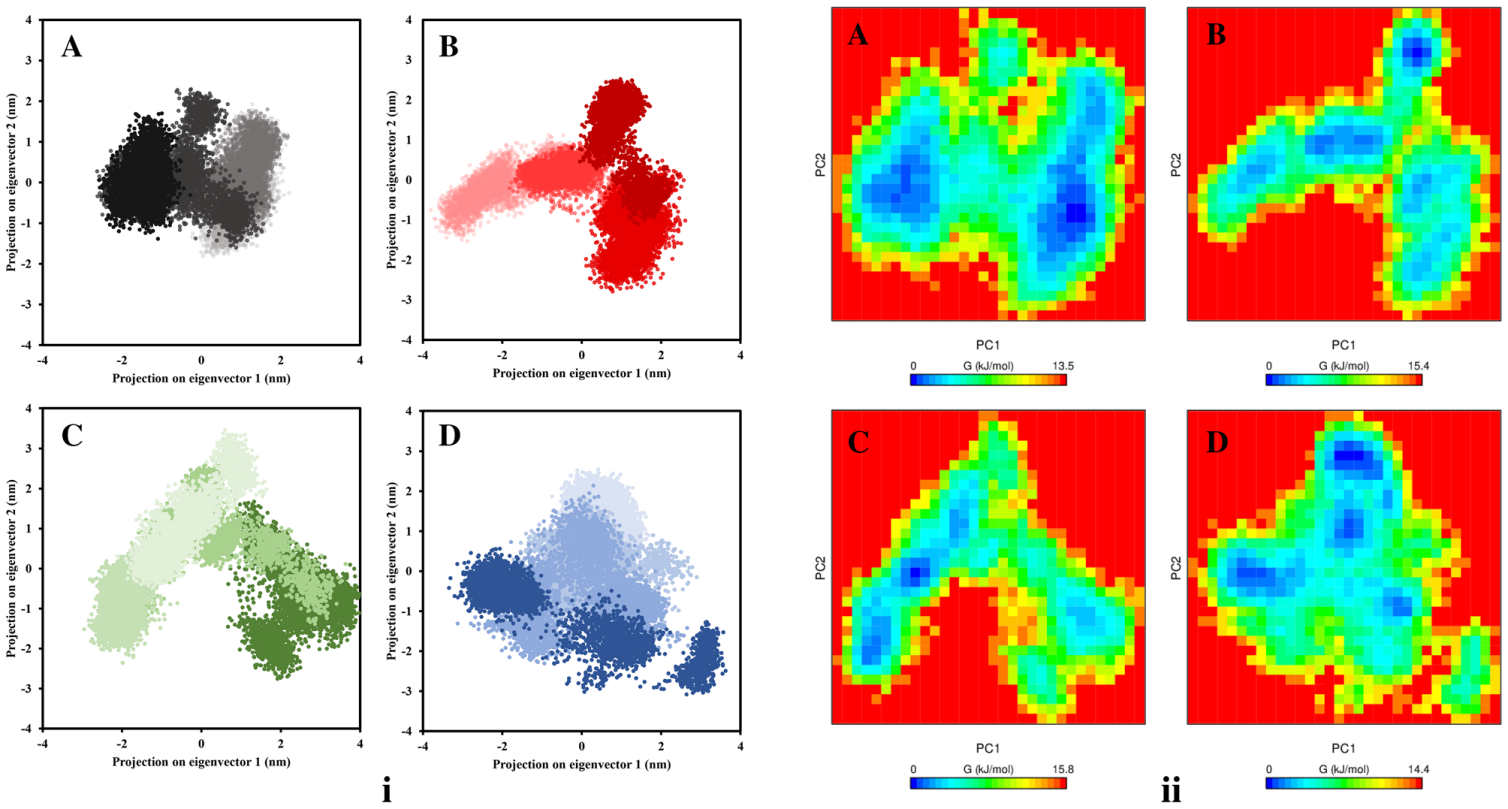
(i) Principal component analysis (PCA) of unbound and bound SRCR5 forms: (**A**) unbound SRCR5 form (black dots), (**B**) SRCR5-brazilin complex (red dots), (**C**) SRCR5-catechin complex (green dot) and (**D**) SRCR5-epicatechin complex (blue dots). (ii) The free energy landscape (FEL) of unbound and bound forms of the SRCR5 receptor: (**A**) unbound SRCR5 form, (**B**) SRCR5-brazilin complex, (**C**) SRCR5-catechin complex and (**D**) SRCR5-epicatechin complex.

Principal component analysis (PCA) was used to investigate the average motion of macromolecule conformations during the MD simulation. PCA was performed for unbound SRCR5 and bound SRCR5 forms (SRCR5-brazilin, SRCR5-catechin, and SRCR5-epicatechin) and structural dynamics were generated utilizing the ‘g_covar’ and ‘g_anaeig’ utility modules of GROMACS software. The first ten eigenvectors for SRCR5 complexes with brazilin, catechin, and epicatechin accumulated 76.41%, 76.95%, and 75.75% of the motions observed, respectively (Fig. S2). All SRCR5 complexes showed similar motions, whereas unbound SRCR5 showed lower motions among all of the complexes (Fig. 3A). The PC1 versus PC2 of FEL revealed that the SRCR5-brazilin complex showed a much more defined and stable cluster at the last snapshot (blue valley) than all other bound SRCR5 receptors (Fig. 3B), while the SRCR5-catechin (Fig. 3C) and SRCR5-epicatechin complexes (Fig. 3D) showed stable clusters at the first snapshot and unstable clusters in phase space.

#### Hydrogen bond analysis

**Figure 4:**
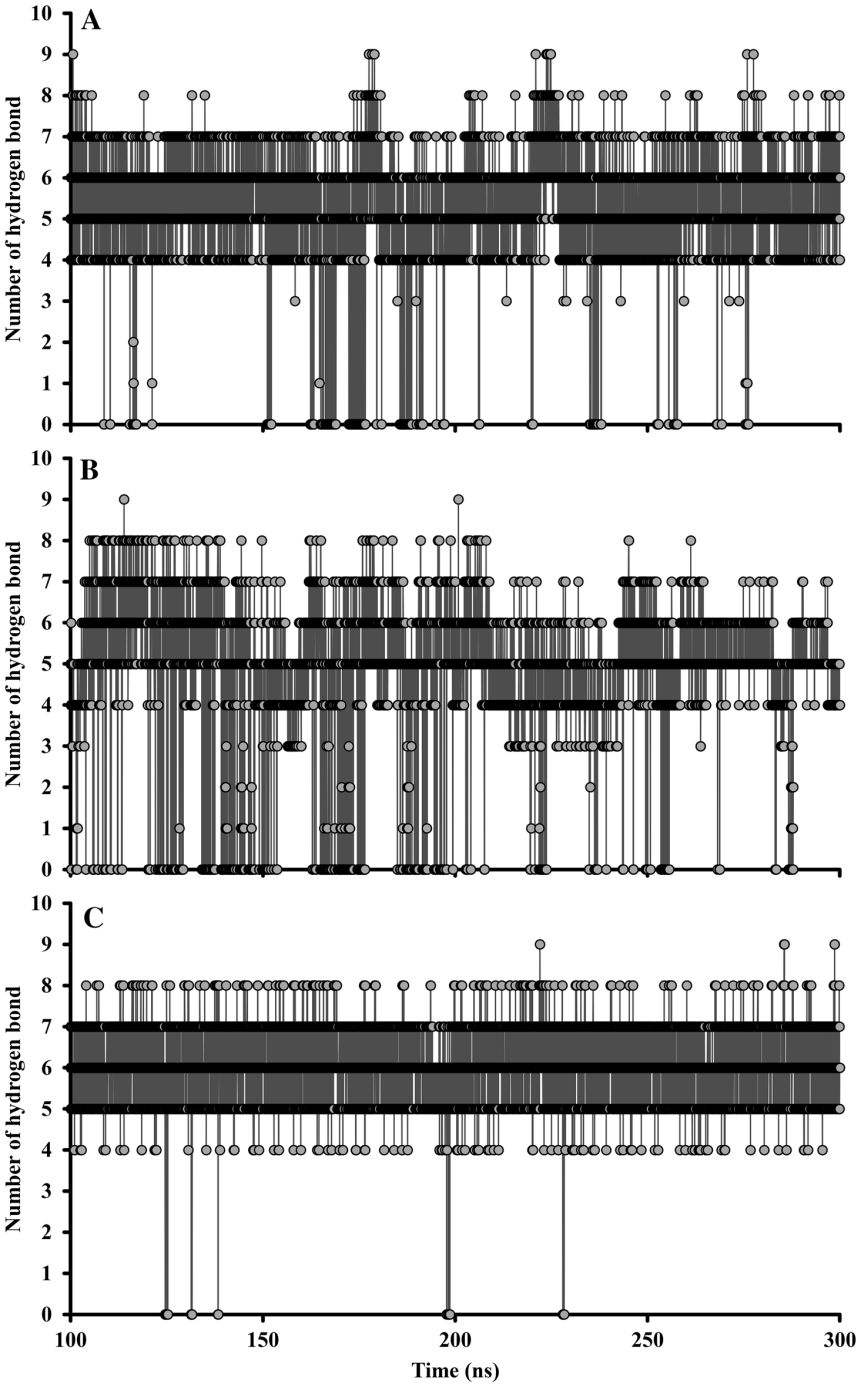
Hydrogen bond analysis of bound SRCR5 forms with three natural compounds: (**A**) SRCR5-brazilin, (**B**) SRCR5-catechin, and (**C**) SRCR5-epicatechin complexes.

Hydrogen bond analysis was performed via the ‘g_hbond’ utilities module of GROMACS software. Bound SRCR5 receptor with inhibitor (brazilin, catechin, and epicatechin) complexes exhibited hydrogen bonding at stable trajectories (100–300 ns). The default parameter was determined based on cutoffs to 0.35 nm of donor–acceptor for distance and 30 degrees of hydrogen-donor-acceptor for angle. Brazilin and epicatechin bound to the SRCR5 receptor showed higher hydrogen bonding stability than catechin bound to the SRCR5 receptor (Fig. 4). The average numbers of hydrogen bonds within SRCR5-brazilin, SRCR5-catechin, and SRCR5-epicatechin were 5.11 ± 1.45, 4.62 ± 1.86, and 6.06 ± 0.70, respectively.

**Figure 5:**
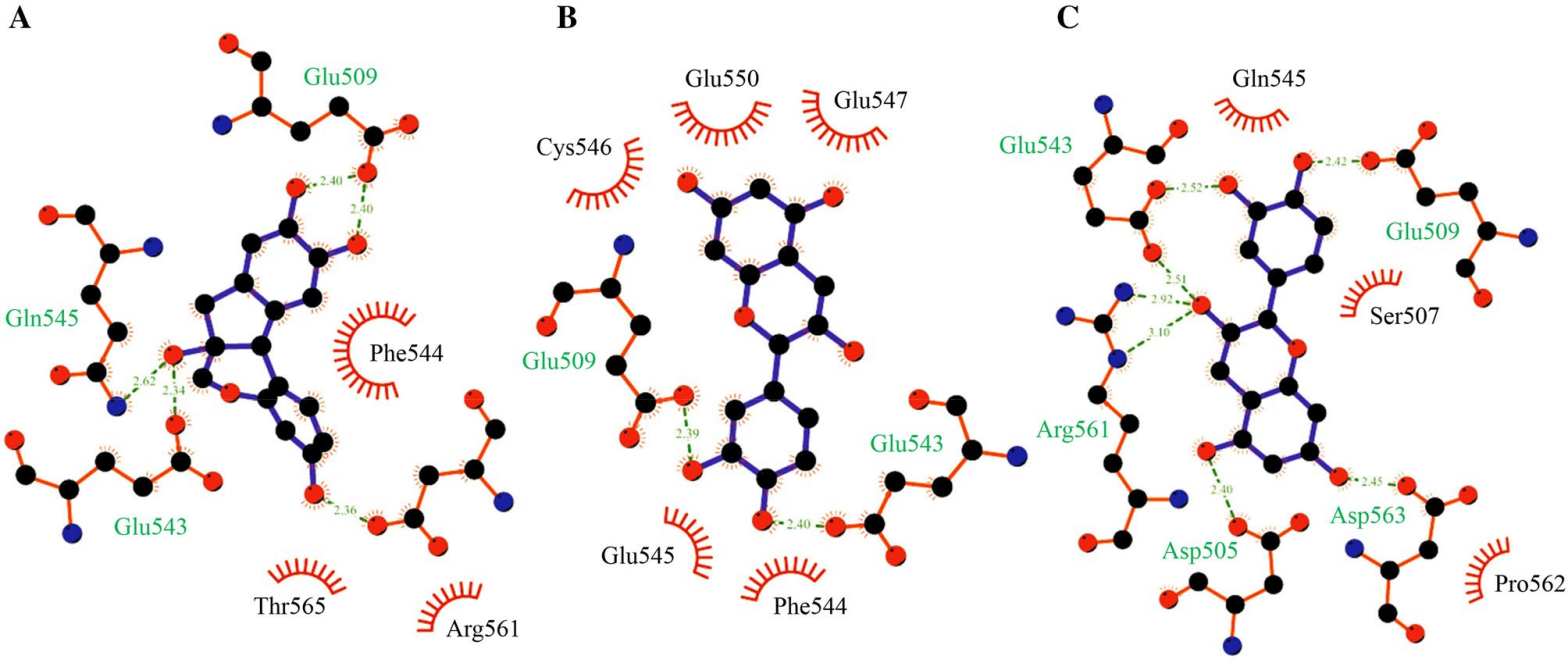
Hydrophobic interactions and hydrogen bonding of bound SRCR5 forms with three natural compounds: (**A**) SRCR5-brazilin, (**B**) SRCR5-catechin, and (**C**) SRCR5-epicatechin complexes.

The last snapshot of all MD complexes was converted to pdb file format to visualize the protein‒ligand interactions using LigPlot^+^ software. Ligplot^+^ software can analyze both hydrogen bonds and hydrophobic interactions. Figure 5A shows that the SRCR5-brazilin complex was found to have amino acid residues Phe544, Arg561, and Thr565 as hydrophobic interactions as well as Glu509, Glu543, Gln545, and Asp563 with hydrogen bonding. The SRCR5-catechin complex was found to have amino acid residues Phe544, Glu545, Cys546, Glu550, and Glu547 as hydrophobic interactions as well as Glu509 and Glu543 with hydrogen bonding (Fig. 5B). The SRCR5-epicatechin complex was found to have amino acid residues of Ser507, Glu545, and Pro562 as hydrophobic interactions as well as Ser505, Glu509, Glu543, Arg561, and Asp563 with hydrogen bonding (Fig. 5C).

## Molecular mechanics calculations

### Binding free energy analysis

To address the binding properties between the SRCR5 receptor and the three compounds (brazilin, catechin, and epicatechin), complexes were calculated by using the molecular mechanics Poisson-Boltzmann surface area (MM-PBSA) approach and are shown in Table 1. The MM-PBSA calculation results showed that the binding free energies between the SRCR5 receptor were − 15.71, − 9.21 and − 11.58 kcal/mol for brazilin, catechin, and epicatechin, respectively, which indicates that the SRCR5-brazilin complex is more stable than the SRCR5-catechin and SRCR5-epicatechin complexes.

**Table 1:** Binding energy components of bound SRCR5 forms obtained from the molecular mechanics Poisson-Boltzmann surface area method (MM-PBSA).

Complexes	*E*_*VdW*_	*E*_*Elec*_	*E*_*Polar*_	*E*_*Non-polar*_	Binding energy
SRCR5-Brazilin	− 6.30	− 24.06	34.43	− 19.78	− 15.71
SRCR5-Catechin	− 3.70	− 27.72	42.82	− 20.61	− 9.21
SRCR5-Epicatechin	− 2.16	− 28.11	39.84	− 21.15	− 11.58

All energy terms are represented in kcal/mol unit.

### In vitro inhibition of PRRSV infection of MARC-145 cells

**Figure 6:**
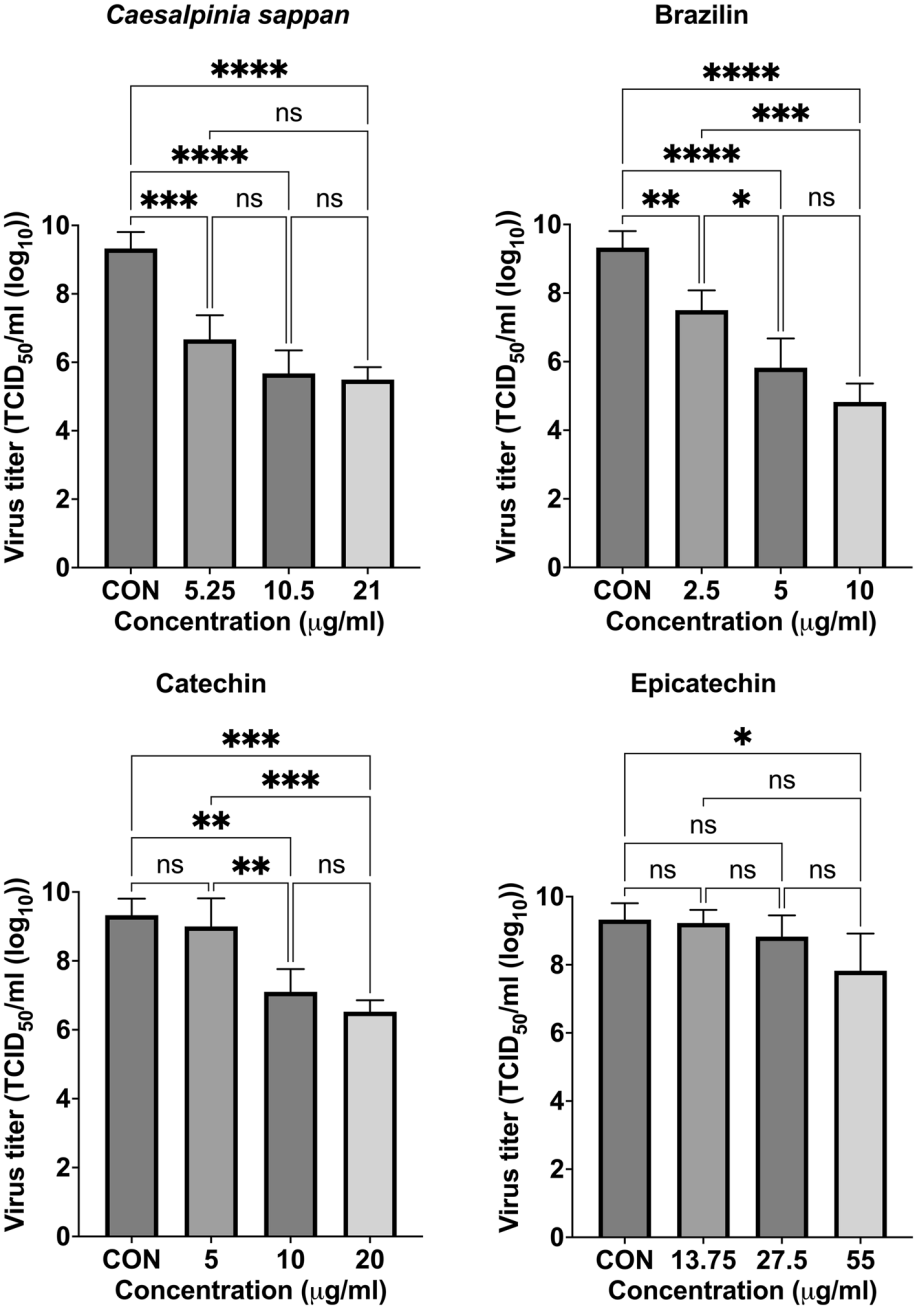
Virus titer of *Caesalpinia sappan* extract, brazilin, catechin, and epicatechin on inhibition of viral infection against PRRSV in MARC-145 cells as measured by IPMA assay. *Caesalpinia sappan* extract, brazilin, catechin, and epicatechin were combined with PRRSV 1 h then inoculate to MARC-145 cell inoculation. The data (mean ± SD) are indicative of four independent replicates. To compare the means of samples with differing concentrations, Tukey’s test was used to assess statistical significance. CON, control; ns, not significant; *P < 0.05; **P < 0.01; ***P < 0.001; ****P < 0.0001.

**Figure 7:**
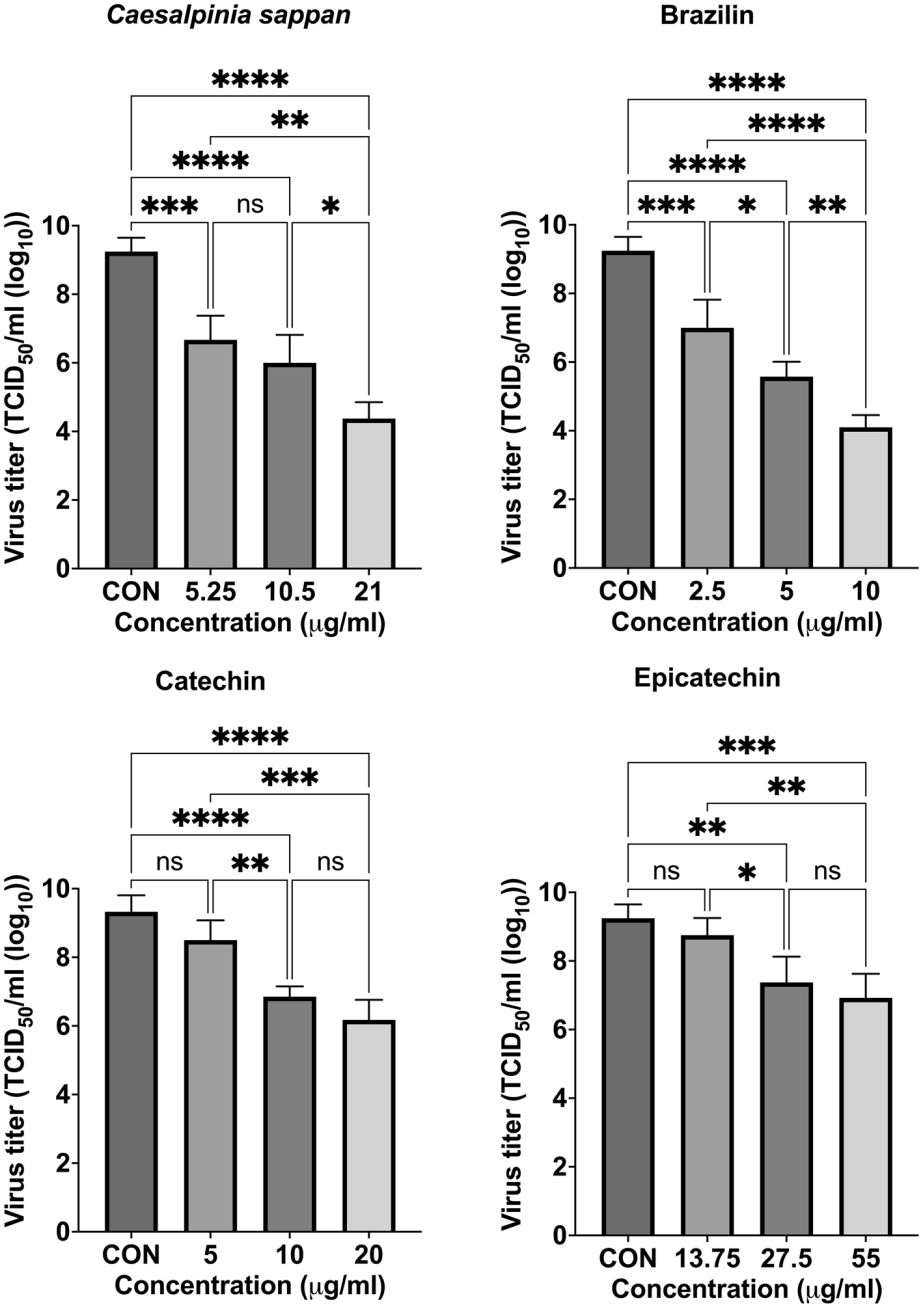
Virus titer of *Caesalpinia sappan* extract, brazilin, catechin, and epicatechin on inhibition of viral infection against PRRSV in MARC-145 cells as measured by IPMA assay. MARC-145 cells were treated with *Caesalpinia sappan* extract, brazilin, catechin, and epicatechin for 1 h, then inoculated with PRRSV (MOI = 1). The data (mean ± SD) are indicative of four independent replicates. To compare the means of samples with differing concentrations, Tukey’s test was used to assess statistical significance. CON, control; ns, not significant; *P < 0.05; **P < 0.01; ***P < 0.001; ****P < 0.0001.

**Figure 8:**
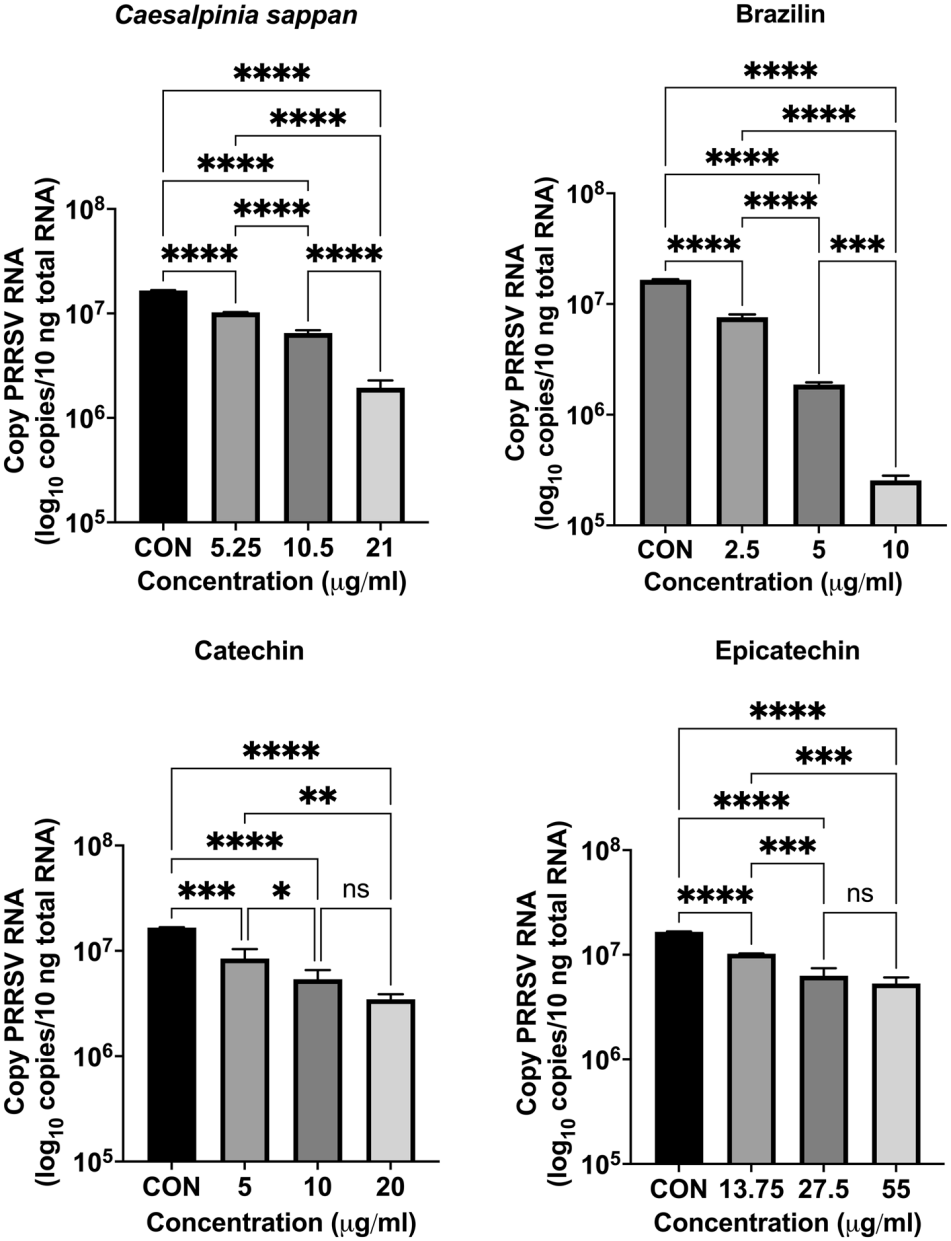
Intracellular PRRSV RNA release evaluated by real-time PCR of MARC-145 cells treated with *Caesalpinia sappan* extract, brazilin, catechin, and epicatechin combined with PRRSV 1 h prior to MARC-145 cells inoculation.The data (mean ± SD) are indicative of three independent replicates. To compare the means of samples with differing concentrations, Tukey’s test was used to assess statistical significance. CON, control; ns, not significant; *P < 0.05; **P < 0.01; ***P < 0.001; ****P < 0.0001.

**Figure 9:**
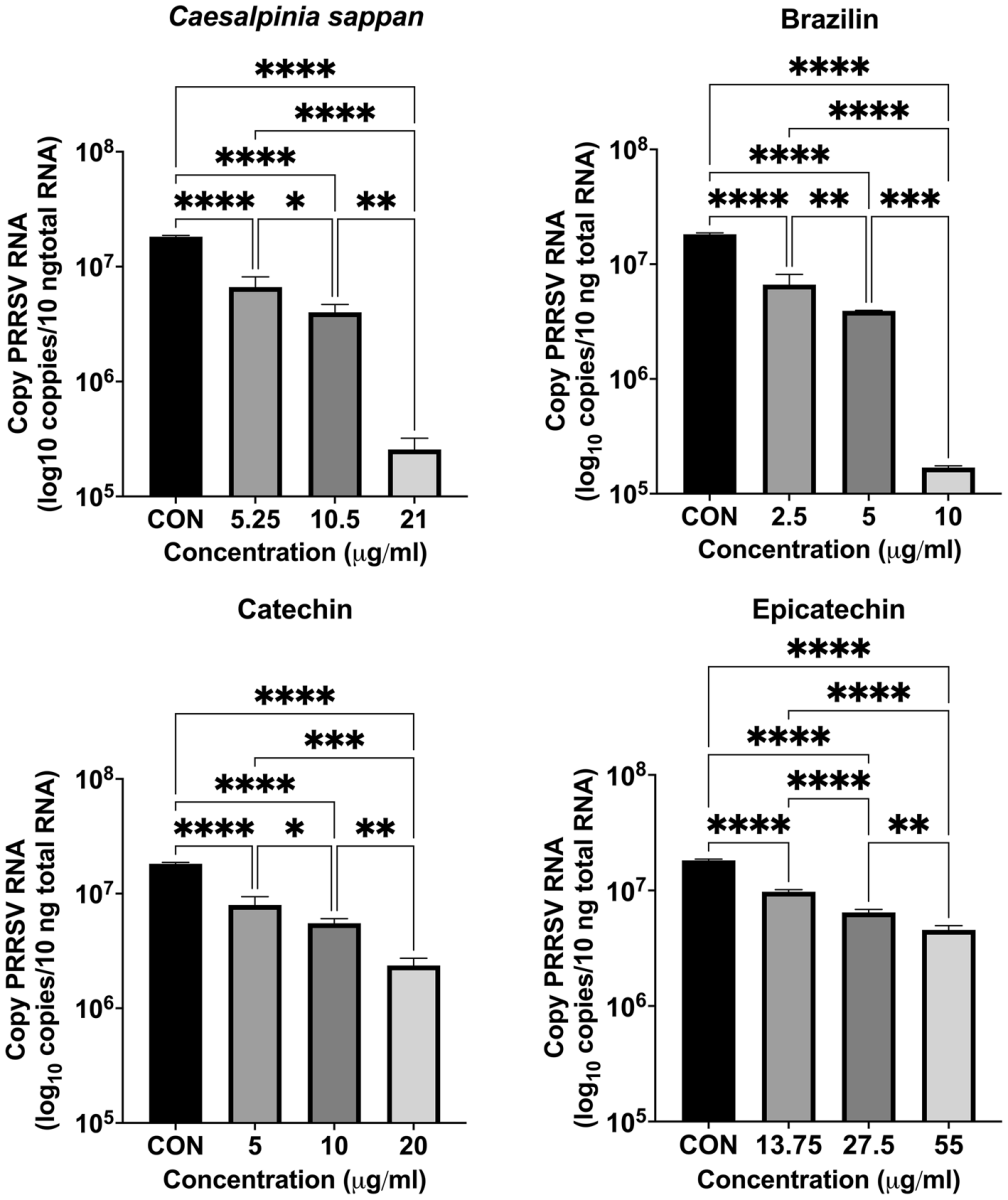
Intracellular PRRSV RNA release determined by real-time PCR of MARC-145 cells treated with *Caesalpinia sappan* extract, brazilin, catechin, and epicatechin for 1 h, then inoculated with PRRSV (MOI = 1). The data (mean ± SD) are indicative of three independent replicates. To compare the means of samples with differing concentrations, Tukey’s test was used to assess statistical significance. CON, control; ns, not significant; * *P* < 0.05; ** *P* < 0.01; *** *P* < 0.001; ****P < 0.0001.

To demonstrate the inhibitory effects of the samples on the interaction with the CD163-SRCR5 domain, we performed in vitro experiments in MARC-145 cells. This study treated PRRSV with various doses of samples whose CC_50_ values (Supplemental Data 1) were calculated to have no effect on the proliferative activity of MARC-145 cells. First procedure, in comparison to the control group (9.33 TCID_50_/mL), the maximum dosage of crude CS extract (21 µg/ml) inhibited PRRSV infection at a viral titer of 4.77 TCID_50_/mL at 24 hpi at a considerably higher level (Fig. 6). However, of the active components of CS extract, only brazilin suppressed PRRSV infection in the MARC-145 model. Brazilin at a concentration of 10 µg/ml significantly inhibited PRRSV infection by 10^–4^^5^ TCID_50_/mL compared to the control group (virus titer, 4.83 vs. 9.33 TCID_50_/mL, respectively). Meanwhile, the second procedure show the same outcomes were seen when MARC-145 cells were used to treat samples before PRRSV inoculation (Fig. 7). Brazilin at a concentration of 10 µg/ml significantly inhibited PRRSV infection in MARC-145 cells by 10^–5^^15^ TCID_50_/mL compared to the control group (virus titer = 4.10 vs. 9.25 TCID_50_/mL, respectively). The RNA copies of PRRSV in MARC-145 cells of CS and brazilin as assessed by RT‒qPCR were compatible with the viral titer results (Fig. 8). There was an effectively decreased number of PRRSV RNA copies when cells were treated with CS and brazilin at all concentrations compared with the control group. At 10 µg/ml, brazilin was found to be a effective compound that could prevent PRRSV infection in MARC-145 cells by decreasing the number of PRRSV RNA copies within the cells. Regarding catechin, doses of 10 and 20 g/ml effectively suppressed PRRSV compared to the control group. RT‒qPCR confirmed the efficiency of catechin in preventing PRRSV infection. However, in MARC-145 cells, epicatechin was ineffective against PRRSV infection. Only the highest concentration (55 g/ml) of epicatechin displayed antiviral activity against PRRSV infection, while the RNA copies of PRRSV were decreased. However, the second procedure when the sample was incubated with MARC-145 before being infected with PRRSV, it was discovered that CS extract and brazilin have the effectively to reduce the PRRSV RNA copy than catechin and epicatechin (Fig. 9).

## Discussion

Medicinal plants are potential alternative antiviral sources that could be used to protect pigs. Antiviral activity is one of the properties possessed by the bioactive chemicals within medicinal plants. CS is a medicinal plant that contains bioactive compounds with a variety of characteristics. Various previous reports have stated that brazilin is the major element of CS^26–28^. To date, however, there have been no published data on brazilin's antiviral activity against pig infections. Previous reports showed that crude CS had efficient antiviral activity against PRRSV in MARC-145 cell models^17^. Our prior research separated crude CS extract into 6 fractions and discovered that fraction 1 demonstrated anti-PRRSV activity in vitro; this fraction included components such as brazilin, catechin, and epicatechin^11^. Until now, there has been ambiguity about the mechanisms of the active compounds from CS that exhibited anti-PRRSV activities. Therefore, the goal of our study was to determine the efficacy of the bioactive components contained in CS extract, including brazilin, catechin, and epicatechin, as well as the crude extract of CS against PRRSV. The capacity of the bioactive chemicals to prevent PRRSV by inhibiting virus infection was predicted based on prior in vitro studies and computational determinations.

Porcine reproductive and respiratory syndrome (PRRS) caused by PRRSV is a significant swine disease worldwide. Blocking viral infection in the binding or attachment step and binding of PRRSV to the receptor on the SRCR5 position of the CD163 protein may help to decrease the PRRSV pandemic. Information on drug or inhibitor binding to the SRCR5 receptor is lacking. Therefore, we investigated computational approaches for proposing natural compounds that can inhibit PRRSV infection via in silico and in vivo methods. These results from using combination methods revealed that brazilin, a major compound from *Caesalpinia sappan* L. extract, could block PRRSV attachment on the cell surface receptor of porcine CD163 and prevent virus infection.

To identify the binding affinity of compounds that bind to the SRCR5 receptor, we used molecular docking and molecular dynamics simulations to dramatically stabilize the protein structure and molecular mechanics calculations (MM-PBSA) to estimate the binding free energies between the SRCR5 receptor and natural compounds as inhibitors. Brazilin was a more potent, stronger inhibitor against PRRSV infection than catechin and epicatechin.

The molecular interactions of the SRCR5 receptor and the three compounds were preliminarily observed in hydrogen bonding (Ser504, Asp505, Ser507, Glu543, Phe544, Gln545, and Cys546) and hydrophobic interactions (Ser504, Phe506, Ser507, Ala510, and Gln545) via molecular docking studies. The conserved amino acid residues and binding affinities had similar profiles and values. Therefore, based on the molecular docking technique, the potency of compounds bound to the SRCR5 receptor could not be separated.

Moreover, the analyses of protein‒ligand interactions showed that all bound SRCR5 forms with the major compound extract from *Caesalpinia sappan* L. were involved in hydrogen bonding, hydrophobic interactions and π-interactions with the binding site residues of the SRCR5 receptor. Based on the computational results, the interactions of the SRCR5-brazilin complex suggested that the amino acid residues Asp505, Glu509, and Arg561 could interact with the 1,2-dihydroxybenzene fragment of brazilin via hydrogen bonding, while Phe544, Glu543, and Cys546 interacted with the aromatic fragment of brazilin via π-interactions. These SRCR5-brazilin complexes still displayed a salt-bridge interaction structure between Arg561 and Asp505 on the SRCR5 surface and stabilized between residues 499–508 and 559–573 of the two loop regions. In addition, the molecular mechanics calculation suggested that the van der Waals, electrostatic and nonpolar solvation energies of SRCR5-brazilin showed favorable contributions and might play a role in maintaining the stability of protein–ligand binding, while the polar solvation energy showed positive values.

Therefore, we decided to test the in vitro antiviral activity of the active compound from CS extract against PRRSV. Interestingly, CS crude extract high efficiently inhibited PRRSV infection to MARC-145 cells by half compared with the control group. Moreover, brazilin was the only active compound that could inhibit PRRSV infection of MARC-145 through a virus titer assay, with almost the CS crude extract efficiency. In accordance with the virus titer, RT‒qPCR indicated that brazilin suppressed PRRSV copies in MARC-145 cells in a highly effective manner. This in vitro antiviral activity study found that CS extract and its active compounds did not reduce ability of viruses to infect cells. While, the compounds were mixed and incubated with PRRSV for 1 h before being inoculated into MARC-145 cells, the cells became infected. However, after the compounds were incubated for 1 h with MARC-145 cells before adding PRRSV, it was found that CS extract and its compounds could inhibit the infection of PRRSV into cells. However, after the compounds were incubated for 1 h with MARC-145 cells before adding PRRSV, it was found that CS extract and its compounds could inhibit the infection of PRRSV into cells. Therefore, this in vitro study confirmed the result of the computational prediction that simulated the mechanism of an active compound via SRCR5 receptor on porcine CD163, especially brazilin which high efficiency inhibits the PRRSV infection.

Brazilin is the main homoisoflavonoid constituent found in CS heartwood^20^. Jung et al.^29^ reported that brazilin was the principal component of the most active fraction obtained fter sequential purification of *C. sappan* extracts via Sephadex and silica gel chromatography and yielded a near-homogenous HPLC peak of approximately 1.15%. However, until now, there have only been limited studies that reported that brazilin from CS can have antiviral activity. Liu et al.^24^ reported that brazilin from CS was efficient at inhibiting influenza virus (H3N2) in vitro. Additionally, in a recent report from Laksmiani et al.^30^, in an in silico model, brazilein and brazilin formed stronger bonds with ACE2, the receptor of SARS-CoV-2, indicating that these compounds exhibit strong affinity for the target protein that plays a role in coronavirus infection. This is the first study to demonstrate the antiviral efficacy of brazilin from CS, specifically the partial suppression of virus infection by PRRSV via the SRCR5 protein receptor in MARC-145 cells. The combination of in silico and in vivo methods may elucidate new findings in drug discovery.

## Materials and methods

### Computational methods

#### Ligand and protein preparations for molecular docking studies

Three structural compounds, brazilin, catechin, and epicatechin, as natural products, were retrieved from the PubChem database^31^ with IDs 73384, 355889196, and 182232, respectively. The three-dimensional structures of the natural compounds were optimized using the steepest descent algorithm in the MMFF94 force field via the Avogadro^32^ program before being subjected to AutoDockTools^33^ software under MGLTools version 1.5.7 for ligand preparation. The crystal structure of scavenger receptor cysteine-rich domain 5 (SRCR5) from porcine CD163 (PDB ID 5JFB)^8^ was retrieved from the RCSB protein databank (https://www.rcsb.org/) and solved by the X-ray diffraction method with a resolution of 2.0 Å. This SRCR5 receptor was removed from the cofactors and carefully checked for the missing residue before being subjected to MGLTools software for protein preparation.

#### Molecular docking studies

Molecular docking was used to predict the ligand pose to the protein receptor, and preliminary docking data were observed as protein‒ligand interactions. Key residues for pointing to the grid box followed previous publications^8,34,35^. Briefly, the key residues (Cys566, Thr565, Arg561, Trp540, and Ile539) of the SRCR5 receptor play important roles in PRRS virus infection. Therefore, a grid size of 40 × 40 × 40 points was set up to cover the key binding site residues of X, Y, and Z, respectively, with a grid spacing of 0.375 Å. The grid center was assigned dimensions of − 12.064, 6.239, and 1.012 for x, y, and z, respectively. The exhaustive parameter was set to 100 for the possible ligand conformation. Molecular docking of the three natural compounds was performed using AutoDock Vina^36^ software (The Scripps Research Institute, La Jolla, CA, USA) on the Linux platform.

#### Molecular dynamics simulations

Molecular dynamics simulations were used to eliminate biased conformations of the ligand bound to the protein after passing the molecular docking studies. The best poses of each of the three natural compounds were chosen based on the following criteria: (1) binding energy range and (2) the ligand can interact with key residues. The SRCR5 protein was generated for topology and Cartesian coordination using the GROMACS software suite^37,38^. Briefly, molecular dynamics simulations were carried out using GROMACS version 4.6.3. The topology and parameters for the protein were generated using the GROMOS 54A7 force field^39^. The topology and parameters for the ligand were generated using the ATB server^40^. Each protein‒ligand complex was merged inside a cubic simulation box with a 10 nm distance to the edges. Solvation was performed using a single point charge (SPC) water model, and counter ions (NaCl) were added to achieve a molarity of 0.15 M for neutralization of the system. Each system was minimized using the steepest descent energy minimization algorithm with an energy step size of 0.01 kJ/mol without constraints until a tolerance limit of 1000 kJ/mol/nm was reached as an energy minimization step, followed by 1000 ps of equilibration in both an NVT ensemble and an NPT ensemble with position restraint of ligand as an equilibration step. The temperature and pressure of each system were set at 300 K and 1 bar, which were controlled by a Berendsen thermostat and a Parrinello-Rahman barostat, respectively. Long-range electrostatic interactions were managed using the particle mesh Ewald algorithm^41^. During the simulations, the lengths of all bonds containing hydrogen atoms were constrained utilizing the LINCS algorithm^42^. An integration step of 2 fs was used. Each system was produced at production for 300 ns, and snapshots were saved every 10 ps for further analysis.

#### Molecular mechanism calculation

The molecular mechanics/Poisson-Boltzmann surface area (MM-PBSA) method was used to estimate the binding free energy of protein–inhibitor complexes during the MD simulations and followed by Kumari^43^. In this study, the total binding energies of the wild-type SRCR5 to bind with the selected natural products brazilin, catechin, and epicatechin were calculated for different component terms, such as molecular mechanics, polar solvation, and nonpolar solvation energies, using the g_mmpbsa script. Generally, the binding free energy $(\Delta {G}_{binding})$ was defined (Eq. 1) as

1 $$ \Delta G_{binding} = G_{complex} - \left( {G_{protein} + G_{ligand} } \right) $$

2 $$ \Delta G_{x} = \left( {\left\langle {E_{MM} } \right\rangle - TS + \left\langle {G_{solvation} } \right\rangle } \right) $$

3 $$ \Delta E_{MM} = \Delta E_{Elec} + \Delta E_{vdW} + E_{bonded} $$

4 $$ \Delta G_{solvation} = \Delta G_{polar} + \Delta G_{nonpolar} $$

5 $$ \Delta G_{nonpolar} = \gamma SASA + \beta $$

where $\Delta {G}_{complex}$, ${G}_{protein}$, and ${G}_{ligand}$ are the total free energies of the protein‒ligand complex, protein and ligand, respectively, in the solvent system. Therefore, the free energy for each individual component is shown in Eq. (2). ${G}_{protein}$ is the total free energy of the protein and ligand, and ${G}_{ligand}$ is the total free energy of the protein, where the enthalpy change $(\Delta H)$ is computed as the sum of changes in the gas-phase energy $(\Delta {E}_{MM})$ and solvation free energy $(\Delta {G}_{solvation})$ averaged over a conformational ensemble generated by MD simulations. $(\Delta {E}_{MM})$ can be denoted by the following formula: $(\Delta {G}_{solvation})$ is the sum of the polar solvation free energy $(\Delta {G}_{polar})$ and the nonpolar solvation free energy $(\Delta {G}_{nonpolar})$. $(\Delta {G}_{polar})$ is estimated by solving the Poisson-Boltzmann equation. The nonpolar solvation term is calculated from the solvent-accessible surface area (SASA) model using the formula (Eq. 5): where γ and β are empirical constants for 0.00542 kcal/(mol Å^2^) and 0.92 kcal/(mol Å^2^), respectively. The stable snapshots (250–300 ns) were separated from the MD trajectories for MM-PBSA calculation.

#### Data analysis and graphic preparation

The MD trajectories and structures of each system were interpreted using the utilities of GROMACS software and visualized structural representation by using Visual Molecular Dynamics (VMD) software^44^, Accelrys Discovery Studio Visualizer 4.0 (Accelrys Software Inc.) and LigPlot^+^^45^ version 2.2.5. SigmaPlot 12.5 (Systat Software, San Jose, CA) was used to generate all plots of the various parameters.

### In vitro* analysis*

#### Samples

Powdered CS heartwood was purchased from Chakkrawatherb Co., Ltd. (Bangkok, Thailand). The report of the Integrated Taxonomic Information System (ITIS) contains taxonomic data about sappan (taxonomic serial number: 506349). The powder (500 g) was macerated in 95% ethanol (3 × 28 L) at room temperature for 72 h, and the extraction process was repeated twice. The combined crude ethanolic extracts were filtered through Whatman No. 1 filter paper. At a temperature of 40 °C, the solvent was completely evaporated using a rotary evaporator. The extracts were kept at 20 °C until further usage. Brazilin, catechin, and epicatechin were purchased from Sigma-Aldrich Co (St Louis, MO, USA).

#### Cells and virus

MARC-145 cells were grown in Dulbecco's modified Eagle’s medium (DMEM) supplemented with 10% fetal bovine serum (Gibco) and 1% penicillin/streptomycin and incubated at 37 °C in 5% CO_2_ in a humidified incubator. PRRSV (VR2332 North American genotype) was propagated in MARC-145 cells, and the virus was titrated using IPMA and then stored at -80 °C. The virus titer was determined and expressed as TCID_50_ according to the Reed–Muench method^46^.

#### Cell cytotoxicity

The MTT [3-(4,5-dimethyl-2-thiazolyl)-2,5-diphenyl-2H-tetrazolium bromide] assay was used to determine the effect of the CS extract fractions on MARC-145 cell viability. Briefly, MARC-145 cells were seeded into 96-well plates at a density of 5000 cells/well and incubated in a 5% CO_2_ atmosphere at 37 °C for 24 h. When cells had at least 90% confluence, the medium was removed and replaced with a medium containing two-fold serial dilutions of the samples. Medium without plant extract was used as a control. The plates were incubated at 37 °C under a 5% CO_2_ atmosphere for 72 h. Thereafter, the medium was removed, and 20 μL of freshly prepared 5 mg/ml MTT solution was added to each well and incubated again at 37 °C for 4 h. After that, the medium was removed and replaced with 150 μL DMSO to dissolve the crystals, and the plates were shaken for 5 min to dissolve any air bubbles before measuring the MTT signal at an absorbance of 550 nm. The results are represented as CC_50_, which was described as the extract concentration that reduced the cell viability by 50% when compared to untreated controls^47^.

#### Inhibition of viral infection assay

The inhibition of viral infection assay was conducted using two distinct procedures (before and after incubation of CS extract, brazilin, catechin, and epicatechin with MARC-145 cells)^48^. First procedure, PRRSV at a MOI of 1 was mixed with medium containing samples at the cytotoxicity test concentration and two lower concentrations in a twofold dilution and then incubated at 37 °C for 1 h. The control was a medium containing PRRSV mixed with 1% DMSO. MARC-145 cells were then infected with a mixture of PRRSV and the samples, including the control, at a concentration of 5000 cells per well in 96-well plates and incubated at 37 °C for one hour. The media was then discarded and replaced with new medium containing 2% FBS. After 24 h, the supernatant was collected for determine virus titer.

Second procedure, 5,000 MARC-145 cells per well were incubated with the samples at the cytotoxicity test concentration and two lower concentrations in a two-fold dilution at 37 °C for 1 h, then the PRRSV (MOI = 1) was added, and the plate was re-incubated for 1 h. Discard the medium after one hour and replace it with new medium containing 2% FBS. The supernatant was collected 24 h later for virus titer determination.

#### Virus titer

IPMA was used to assess the virus titration as previously described^48^. Briefly, cells were fixed with 100 μl of 4% cold formalin for 15 min at room temperature (RT). The fixed cells were washed with 100 μl phosphate-buffered saline (PBS) once after that and twice with 100 μl of 0.5% PBS Tween-20 (PBST) and then blocked with 100 μL of 1% bovine serum albumin (BSA) in 0.5% PBST for 30 min at RT. Then, the cells were washed. Seventy microliters of anti-PRRSV NC protein monoclonal antibody (Median Diagnostics, Gangwondo, Korea) diluted at a ratio of 1:400 was used to stain cells at RT for 60 min. The cells were washed and incubated again with 50 μl of peroxidase-conjugated AffiniPure Goat Anti-Mouse IgG (H + L) (Jackson Immuno Research, Pennsylvania, USA) at a dilution of 1:600 for 60 min at RT, washed and counterstained with 1,5-diaminopentane (DAP) substrate for 5 min, washed with distilled water and examined under a microscope. Virus titer was determined using the Reed–Muench method^46^, also expressed as TCID_50_, described as diluting a virus required to infect 50% of a given cell culture^49^.

#### Quantitative real-time PCR (RT‒qPCR)

RNA was isolated from PRRSV-infected MARC-145 cells with the PureLink™ RNA Micro Kit (Invitrogen) according to the manufacturer's guidelines. A Nanodrop spectrophotometer was used to quantify the RNA concentrations (Thermo Fisher Scientific). To synthesize cDNA from RNA, iScript Reverse Transcription Supermix for RT‒qPCR (Bio-Rad) was used. Real-time PCR was performed using specific primers for the PRRSV ORF7 gene (forward: 5′ TCAICTGTGCCAGITGCTGG 3′ and reverse: 5′ AAATGIGGCTTCTCIGGITTTT 3′) with a US-PRRSV-specific probe FAM_US_rev (5′ TCCCGGTCCCTTGCCTCTGGA 3′, sense orientation) 5′-labeled with FAM^50^. The real-time was performed on an ABI7500 using the SensiFAST™ Probe NO-ROX KIT (Bioline) following the manufacturer’s instructions. For each experiment, a standard curve was generated using a serially diluted PRRSV standard of 10^3^–10^6^ TCID_50_/ml^12^.

### Statistical analysis

GraphPad Prism 9 (Version 9.4.0, La Jolla, CA, USA) was utilized to conduct a one-way analysis of variance (ANOVA) with Tukey’s post-hoc comparison. The data are reported as the mean and standard deviation, and *P* < 0.05 was considered significant. *P < 0.05; **P < 0.01; ***P < 0.001; ****P < 0.0001.

### Ethics approval and consent to participate

The Chiang Mai University Institutional Biosafety Committee evaluated and authorized all experimental protocols (Approval. No. CMUIBC0663001).

## Conclusion

According to a computational assay, the stability of the SRCR5-brazilin complex is greater than those of the SRCR5-catechin and SRCR5-epicatechin complexes, indicating that brazilin isolated from CS may have the potential to prevent PRRSV infection by blocking the SRCR5 receptor on the CD163 protein. Moreover, IPMA and RT‒qPCR assays revealed that brazilin inhibited PRRSV infection in the same manner. These results provide new insight into the inhibition mechanism of the SRCR5 receptor on porcine CD163 with a natural compound and may aid in the design of new SRCR5 inhibitors for PRRSV infection. Future research may strive to determine the pharmacology of brazilin to produce an antiviral veterinary medication against PRRSV.

## Supplementary Information

Supplementary Information.

## Data Availability

The datasets generated during and/or analysed during the current study are available from the corresponding author on reasonable request.
