# Supplementary material for: Brazilin from Caesalpinia sappan inhibits viral infection against PRRSV via CD163ΔSRCR5 MARC-145 cells: an in silico and in vitro studies
Source: Sci Rep. 2022 Dec 14;12:21595. doi: 10.1038/s41598-022-26206-x (PMC9748407; doi:10.1038/s41598-022-26206-x)
Supplement: Supplementary file 1 — Supplementary Information. [file 41598_2022_26206_MOESM1_ESM.pdf]

Supplementary materials

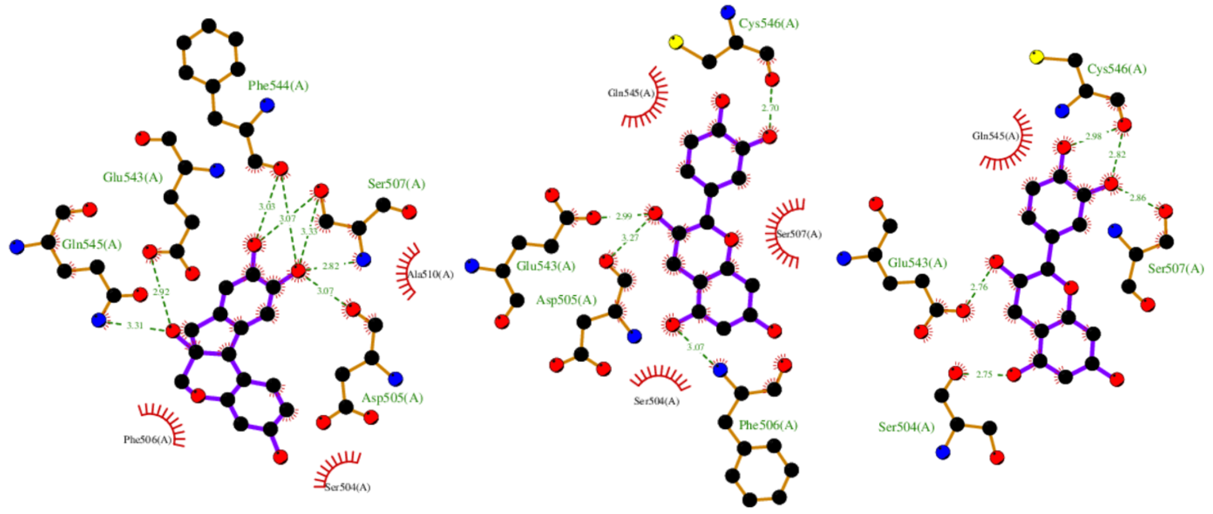

Figure S1 Docking poses of the three natural compounds binding to the binding site of the SRCR5 receptor: A) SRCR5-brazilin, B) SRCR5-catechin, and C) SRCR5-epicatechin complexes.

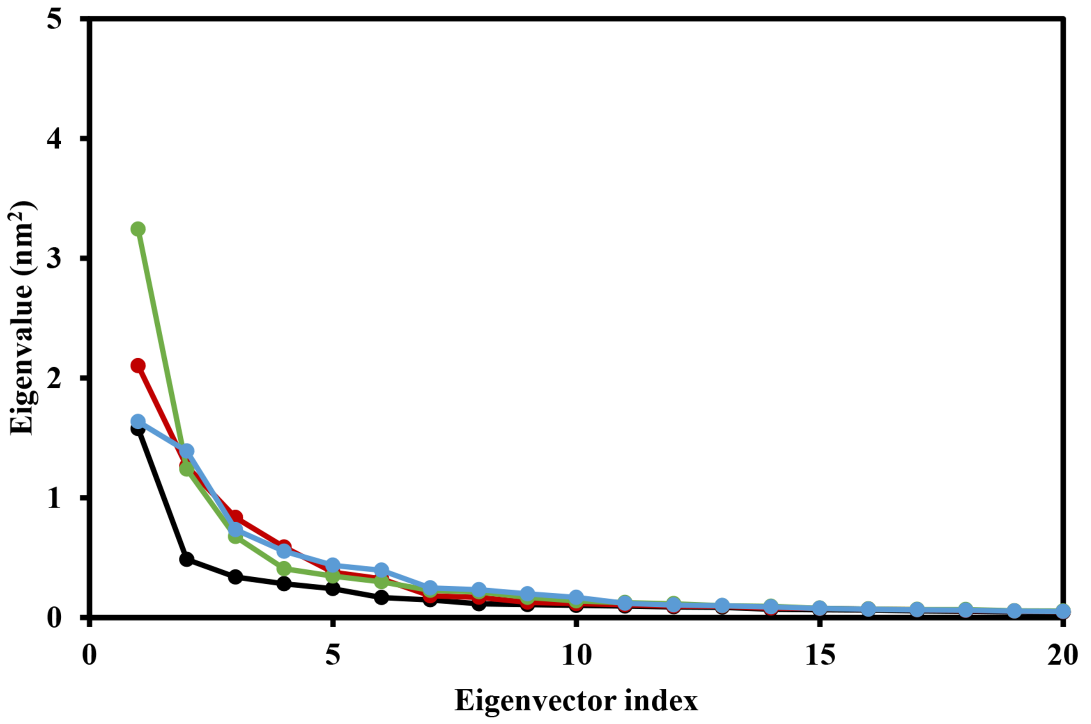

Figure S2 Principal component analysis (PCA) of unbound and bound SRCR5 forms: A) unbound SRCR5 form (black line), B) SRCR5-brazilin complex (red line), C) SRCR5-catechin complex (green line) and D) SRCR5-epicatechin complex (blue line).

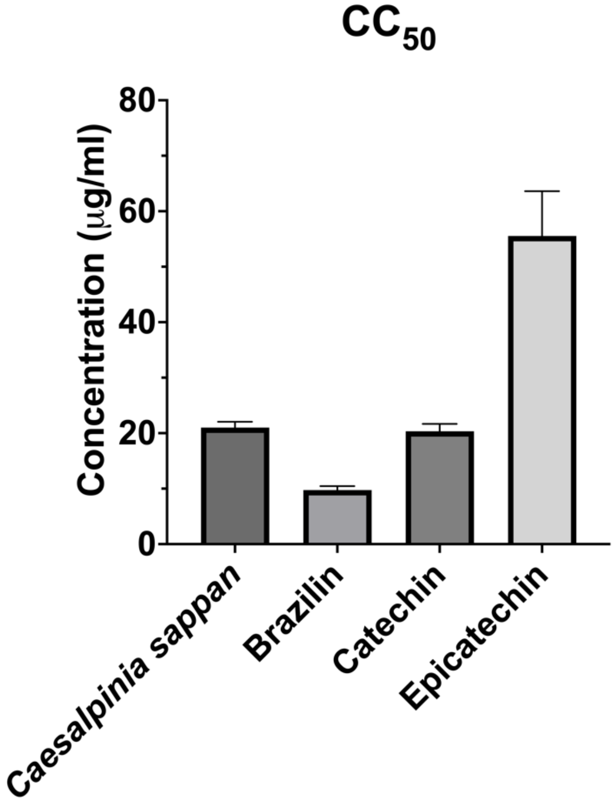

Figure S3 The 3-(4,5-dimethylthiazol-2-yl)-2,5-diphenyl tetrazolium bromide (MTT) assay was used to determine the cytotoxicity of the samples on MARC-145 cells. Prior to the MTT experiment, MARC-145 cells were cultured for 72 hours with varying doses of these samples or with a control containing no samples.

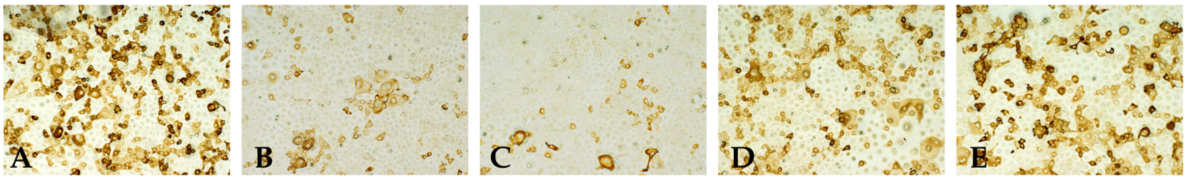

Figure S4 Immunoperoxidase monolayer assay (IPMA) of control (A) CS (B), brazilin (C), catechin (D), and epicatechin (E) on virus infection activity against PRRSV in MARC-145 cells.
